# TCR‐induced alteration of primary MHC peptide anchor residue

**DOI:** 10.1002/eji.201948085

**Published:** 2019-05-27

**Authors:** Florian Madura, Pierre J. Rizkallah, Mateusz Legut, Christopher J. Holland, Anna Fuller, Anna Bulek, Andrea J. Schauenburg, Andrew Trimby, Jade R. Hopkins, Stephen A. Wells, Andrew Godkin, John J. Miles, Malkit Sami, Yi Li, Nathaniel Liddy, Bent K. Jakobsen, E. Joel Loveridge, David K. Cole, Andrew K. Sewell

**Affiliations:** ^1^ School of Medicine Cardiff University Cardiff UK; ^2^ Department of Chemical Engineering University of Bath Bath UK; ^3^ Centre for Biodiscovery and Molecular Development of Therapeutics Australian Institute of Tropical Health and Medicine James Cook University Cairns Queensland Australia; ^4^ Immunocore Ltd. Abingdon UK; ^5^ School of Chemistry Cardiff University Cardiff UK; ^6^ Department of Chemistry Swansea University Swansea UK; ^7^ Systems Immunity Research Institute Cardiff University Cardiff UK

**Keywords:** crystal structure, MART‐1/Melan‐A, peptide–major histocompatibility complex (pMHC), surface plasmon resonance (SPR), T cell: T‐cell receptor (TCR)

## Abstract

The HLA‐A*02:01‐restricted decapeptide EAAGIGILTV, derived from melanoma antigen recognized by T‐cells‐1 (MART‐1) protein, represents one of the best‐studied tumor associated T‐cell epitopes, but clinical results targeting this peptide have been disappointing. This limitation may reflect the dominance of the nonapeptide, AAGIGILTV, at the melanoma cell surface. The decapeptide and nonapeptide are presented in distinct conformations by HLA‐A*02:01 and TCRs from clinically relevant T‐cell clones recognize the nonapeptide poorly. Here, we studied the MEL5 TCR that potently recognizes the nonapeptide. The structure of the MEL5‐HLA‐A*02:01‐AAGIGILTV complex revealed an induced fit mechanism of antigen recognition involving altered peptide–MHC anchoring. This “flexing” at the TCR–peptide–MHC interface to accommodate the peptide antigen explains previously observed incongruences in this well‐studied system and has important implications for future therapeutic approaches. Finally, this study expands upon the mechanisms by which molecular plasticity can influence antigen recognition by T cells.

## Introduction

The interaction between the clonotypic αβ TCR and peptide–MHC (pMHC) governs T‐cell mediated immunity toward pathogens and cancer, and can lead to acute organ transplant rejection and autoimmune disease. TCR–pMHC complex crystal structures, combined with functional experiments, have demonstrated that the TCR complementarity determining regions (CDR) loops engage both the presented peptide and the MHC presentation platform in a generally conserved diagonal orientation, with the TCR‐α chain position over the MHC‐α2 helix and the TCR‐β chain over the MHC‐α1 helix 1. This normal TCR docking orientation typically positions the somatically rearranged CDR3 loops over the center of peptide, and the CDR1 and CDR2 loops over the MHC helices, enabling the TCR to recognize antigen in a peptide‐specific mode 2. Despite this conserved binding geometry, the interaction between the TCR and pMHC can be highly flexible, or even reversed 3, 4, 5. This flexibility is likely to be part of the mechanism that enables T cells to cross‐react with multiple different antigens 6, 7, 8, 9, 10, 11, 12, 13, a property that is required for effective protective immunity 14, 15.

Here, we investigated the Melan‐A (melanoma antigen A)/MART‐1 (melanoma antigen recognized by T‐cells‐1) protein that has been the focus of a number of clinical approaches designed to employ T‐cells for targeting melanoma 16, 17, 18, 19, 20, 21. It has been previously shown that, in the context of HLA‐A*02:01, two overlapping peptides are recognized by CD8^+^ T cells: the MART‐1_27‐35_ nonapeptide AAGIGILTV (A2‐AAG); and the MART‐1_26‐35_ decapeptide EAAGIGILTV (A2‐EAA) 17, 22. However, despite A2‐AAG being the dominant peptide on the surface of tumors 22, most published T‐cell clones recognize A2‐EAA more strongly than A2‐AAG 17, 23, 24. This has fueled a number of investigations that have defined the presentation mode of A2‐AAG, A2‐EAA, and MHC anchor modified “heteroclitic” versions of the peptides, most notably E**L**AGIGILTV (altered residue in bold text) 17, 25, 26. Other investigations have focused on the mechanisms of TCR recognition in this system 24, 27, 28, 29. These studies have shown that the clinically targeted decapeptide, the minority species on tumors 22, is presented in a distinct “bulged” conformation by HLA‐A*02:01, compared to the “stretched” conformation of the nonapeptide 25, 26. Concomitant with the lack of clinical success targeting these antigens, most TCRs recognize the nonapeptide with relatively weak affinities compared to the reported natural range for TCR interactions with cognate pMHC (*K*_D_ = 0.13–270 μM) 30, 31, 32. Structural studies have revealed that two TCRs used in a clinical setting, DMF4 and DMF5, have to make structural compromises when interacting with the nonapeptide 24. In accordance, Blankenstein and colleagues recently showed that T cells, transduced with a clinically relevant TCR, induced rejection of tumors expressing the analog E**L**AGIGILTV, but not native AAGIGILTV, Melan A/MART‐1 epitope 33. Consequently, there is a need to understand the seemingly complex molecular rules that govern T‐cell antigen recognition in this system so that effective therapies can be developed. We previously identified a T‐cell clone (MEL5) 27 that can potently recognize A2‐AAG, consistent with the relatively strong binding affinity (*K*_D_ ≅ 14 μM) for the MEL5‐A2‐AAG interaction 23.

Here, we solved the structure of the MEL5 TCR in complex with A2‐AAG to uncover the mechanisms governing the effective recognition of this tumor antigen. Unexpectedly, we found that MEL5 recognition of A2‐AAG was governed by a “molecular switch” in the peptide. Other published TCRs do not induce this conformational alteration in the A2‐AAG peptide 24, providing a possible reason for why non‐MEL5‐like T cells recognize this melanoma antigen poorly. Importantly, this finding expands our understanding of the mechanisms by which the TCR–pMHC interface can “flex” to accommodate optimal antigen recognition.

## Results

### The MEL5 and α24β17 TCRs induce a peptide anchor residue switch in A2‐AAG

**Figure 1:**
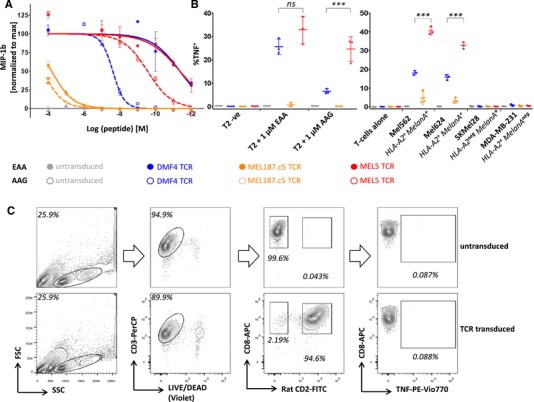
Functional assessment of the response toward HLA‐A*02:01/MART‐1 epitopes mediated by MEL5, MEL187.c5, and DMF4 TCRs. (A) MIP‐1β release, in response to titrated concentrations of MART‐1 decamer (EAAGIGILTV) and nonamer (AAGIGILTV) peptides presented by T2 cells, by T‐cells transduced with the MEL5, MEL187.c5, or DMF4 TCRs. Quantification of secreted MIP‐1β was performed by ELISA. Experiments were performed in duplicate, using PBMCs from three different donors, transduced with MEL5, MEL187.c5, or DMF4 TCRs. Error bars denote SEM from two biological replicates (two independent experiments) using the TCR‐transduced T cells from one representative donor. (B) TNF alpha production in response to MART‐1 decamer and nonamer peptides presented by T2 cell line (left panel) or endogenously processed and presented epitopes by cancer cell lines. Experiments were performed in duplicate, using PBMCs from three different donors, transduced with MEL5, MEL187.c5, or DMF4 TCRs. Mean percentage and SD of TNF^+^ events among viable CD8^+^ (and rat CD2^+^ where applicable) cells from three donors is shown. Two sided Student's *t*‐test; ns, not significant; *p* > 0.05; ***p < 0.001. (C) Gating strategy for assessing the functional response of TCR transduced cells to cancer cells or exogenously supplied agonist peptides. Gating strategy for untransduced (top panel) or TCR transduced (bottom panel) T cells are shown. Rat CD2 is a marker of TCR transduction. Numbers on contour plots indicate the percentage of events in marked gates.

We have previously shown that the MEL5 TCR can bind to A2‐AAG with a stronger affinity (*K*_D_ = 14.2 μM) 23 than other published TCRs (average *K*_D_ = 101 μM; **Table** 1). This approximately sevenfold average difference in TCR affinity is likely to have substantial benefits for recognition of A2‐AAG at the melanoma cell surface 34. To confirm the potential benefits of the MEL5 TCR, we transferred MEL5, MEL187.c5, and DMF4 16 TCRs into primary CD8^+^ T cells for comparison. In order to avoid bias induced by differences in the efficiency of lentiviral transduction, our constructs encoded a surface‐expressed marker gene (rat CD2), which was used to isolate TCR transduced T‐cells and to monitor their purity. MEL5 and DMF4 TCRs showed similar sensitivity to the Melan‐A/MART‐1 decapeptide (EAAGIGILTV) in terms of MIP‐1β secretion; conversely, the sensitivity of MEL5 TCR to the nonapeptide AAGIGILTV was 100× higher than the sensitivity of DMF4 TCR (Fig. 1A). The increased reactivity of MEL5 TCR toward the AAGIGILTV epitope, compared to DMF4 TCR, was further supported by TNF production (Fig. 1B, left panel; Supporting Information Fig. S1A). In line with these findings, MEL5 TCR was capable of mounting a significantly stronger response to HLA‐A*02:01 positive melanoma cell lines than DMF4, while remaining inert to HLA‐A*02:01 or Melan‐A negative cancer cells (Fig. 1B, right panel; Supporting Information Fig. S1B). MEL187.c5 TCR induced only a weak response even to the heteroclitic variant E**L**AGIGILTV (Supporting Information Fig. S2), despite favorable biophysical properties (Table 1). Due to lack of reactivity, we did not include MEL187.c5 TCR in subsequent experiments. The gating strategy is shown in Figure 1C.

**Table 1:** MEL5, MEL187.c, DMF4, DMF5, and α24β17 binding affinities (*K*_D_): A2‐AAG versus A2‐EAA versus A2‐ELA

TCR binding affinity *K*_D_					
Ligand	MEL5	MEL187.c5	DMF4	DMF5	α24β17
A2‐AAG	14.2 ± 0.7 μM 23	94 ± 22 μM 23	170 ± 11 μM 24	40 ± 2 μM 24	26.2 nM
A2‐EAA	8.4 ± 0.2 μM 23	42 ± 0.3 μM 23	nr	nr	0.75 nM 29
A2‐ELA	18 ± 1 μM 27	18 ± 0.1 μM 23	29 ± 4 μM 24	5.6 ± 1.5 μM 24	0.6 nM 29

nr, not reported.

**Figure 2:**
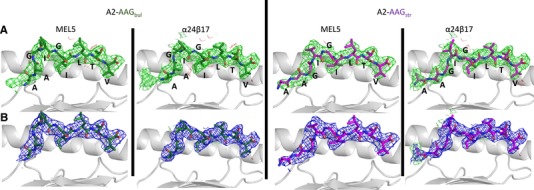
MEL5‐A2‐AAG and α24β17‐A2‐AAG omit map analysis. (A and B) In all panels HLA‐A*02:01 is colored in grey cartoon, with AAGIGILTV presented in the “bulged” conformation (A2‐AAG_bul_) colored in green sticks, or presented in the “stretched” (A2‐AAG_str_) conformation colored in purple sticks. From left to right: first panel is MEL5‐A2‐AAG_bul_, second panel is α24β17‐A2‐AAG_bul_, third panel is MEL5‐A2‐AAG_str_ and last panel is α24β17‐A2‐AAG_str_. (A) Difference electron density omit maps showing a side view of the peptide in each complex in which the model was refined in the absence of the peptide with difference density contoured at 3.0 σ (positive contours colored green, negative contours colored red). (B) 2Fo‐Fc peptide electron density maps at 1 σ (shown in blue) showing a side view of the peptide in each complex. These data were generated from a single data set derived from X‐ray crystallographic analysis.

**Figure 3:**
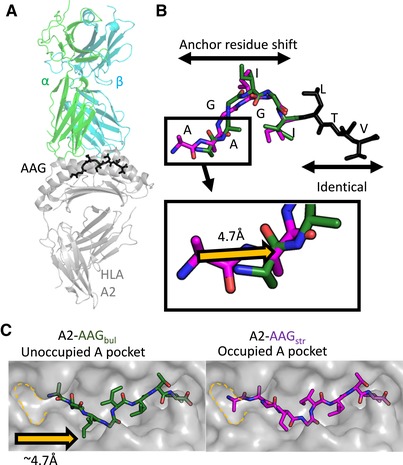
MEL5 and α24β17 TCRs induce a peptide anchor residue switch in A2‐AAG. (A) Overall conformation of the MEL5 (α‐chain green cartoon, β‐chain cyan cartoon) in complex with HLA‐A*02:01 (grey cartoon) presenting the AAG peptide (green, purple, and black sticks). (B) Top: the peptide anchor residue switch in the AAG peptide is depicted with the residues that shift in the AAG_bul_ form in green sticks, and the residues that shift in the AAG_str_ form in purple sticks. Bottom: expanded view of the N‐terminal of the AAG peptide with the shift in position of Cα of alanine residue 1 indicated by the yellow arrow. (C) Top down view of the AAGIGILTV peptide binding to the HLA‐A*02:01 groove (grey) presented in the ‘bulged’ conformation (A2‐AAG_bul_) colored in green sticks, or presented in the “stretched” (A2‐AAG_str_) conformation colored in purple sticks. In bulged form (left), peptide residue Ala1 Cα moves ∼4.7Å compared to the stretched form (right) (highlighted using yellow dashed line) leaving the A‐pocket unoccupied. These data were generated from a single data set derived from X‐ray crystallographic analysis.

The superior recognition of AAGIGILTV peptide and tumor targets by MEL5 TCR suggests that it might make a good clinical candidate for recognizing melanoma cells. To understand the structural basis for this improved recognition, we solved the structure of MEL5‐A2‐AAG using X‐ray crystallography (Supporting Information Table S1). Unexpectedly, we observed partial occupancy in the electron density map of the peptide at the N‐terminus, representing two different conformations, supported by omit map and density analysis (Fig. 2). In one form, the AAG peptide was presented in a “stretched” (A2‐AAG_str_) conformation compared to the “bulged” conformation (A2‐AAG_bul_) observed in the alternative form. To confirm this unusual observation, we investigated the binding mode of a previously published high affinity variant of MEL5, the α24β17 TCR 29. The α24β17 TCR bound to A2‐AAG and A2‐EAA with nanomolar affinity (*K*_D_ = 26.2 and 0.75 nM, respectively; **Table** 1) and the structure of this TCR in complex with A2‐AAG (Supporting Information Table S1) revealed the same partial occupancy in the electron density of the peptide (Fig. 2). The overall binding mode of the MEL5 (Fig. 3A) and α24β17 TCRs in complex with A2‐AAG was similar to the MEL5‐A2‐EAA complex. This was reflected by similar crossing angles of 48° and 42.3° and buried surface areas (BSAs) of ≈2200 and ≈2600 Å for MEL5 and α24β17 TCRs, respectively, in complex with A2‐AAG (Table 2), compared to a crossing angle of 46.9° and BSA of 2366 Å for MEL5‐A2‐EAA 28.

**Table 2:** Summary of co‐complex structures of MEL5‐A2‐AAG and α24β17‐A2‐AAG

Contacts	MEL5‐A2‐AAG_bul_	MEL5‐A2‐AAG_str_	α24β17‐A2‐AAG_bul_	α24β17‐A2‐AAG_str_
TCR/pMHC^a^	8/1/75	8/1/74	11/2/136	11/2/132
TCRα‐pMHC^a^	3/1/31	3/1/30	6/1/58	6/1/55
TCRβ‐pMHC^a^	5/0/44	5/0/44	6/0/68	6/0/77
TCR/peptide^a^	4/0/27	4/0/26	5/0/32	5/0/28
TCR/MHC^a^	4/1/48	4/1/48	6/2/104	6/2/104
BSA^b^ (Å^2^)	1620/655/2275	1625/545/2170	1944/682/2626	1951/662/2613
SC^c^ (%)	53.8/63.4/56.8	53.8/57.9/53.5	73.2/61.1/70.9	73.2/42/62
Crossing angle (°)	48.0	48.0	42.3	42.3

a Number of hydrogen bonds (H‐bond) (<3.4 Å)/salt bridges (<3.4 Å)/van der Waals (vdW) (≤4 Å) contacts calculated with CONTACT program from the CCP4 package 66.

b Buried surface area (BSA) (Å^2^) of TCR–MHC/TCR–peptide/TCR–pMHC calculated with PISA 76.

c Shape complementarity (SC) (%) of TCR–MHC/TCR–peptide / TCR–pMHC calculated with SC program from the CCP4 package 66.

Further inspection of the peptide presentation mode revealed that the conformation observed in the TCR‐A2‐AAG_bul_ structures required an anchor residue shift at the N‐terminus of the peptide, utilizing Ala1 as the primary MHC anchor in the B pocket, rather than Ala2 as in the TCR‐A2‐AAG_str_ structures (Fig. 3B). Indeed, the Cα of Ala1 shifted ≈4.7Å in order to mediate this binding mode in the structures with both WT and enhanced affinity TCRs. This anchor residue shift left the HLA‐A*02:01 A‐pocket unoccupied (Fig. 3C). The only other example in humans where an empty A‐pocket has been observed was in the unnatural, heteroclitic nonapeptide A2‐LAGIGILTV, in which Ala1 was substituted with Leu, an optimal residue for binding to the B‐pocket in HLA‐A*02:01 to artificially induce this binding mode. Substitution of the position 1 Ala residue for Leu enabled the peptide to anchor at residue one, forming a conformation similar to that of A2‐EAA 25. Interestingly, the LAGIGILTV nonapeptide was recognized more strongly than AAGIGILTV by multiple TCRs, demonstrating the importance of a central EAAGIGILTV‐like peptide bulge during T‐cell recognition 25. The switch in anchor residue from Ala2 to Ala1 in the A2‐AAG_bul_ structure enabled peptide residue Gly5 to swing 1.7Å, compared to A2‐AAG_str_, into an identical position compared to A2‐EAA. The ability of MEL5, but not other TCRs, to bind to A2‐AAG in an A2‐EAA‐like conformation probably explains why MEL5 was better able to recognize the immunodominant MART‐1/Melan‐A nonapeptide compared to other published TCRs.

### MEL5 captures A2‐AAG in a different conformation than other TCRs

**Figure 4:**
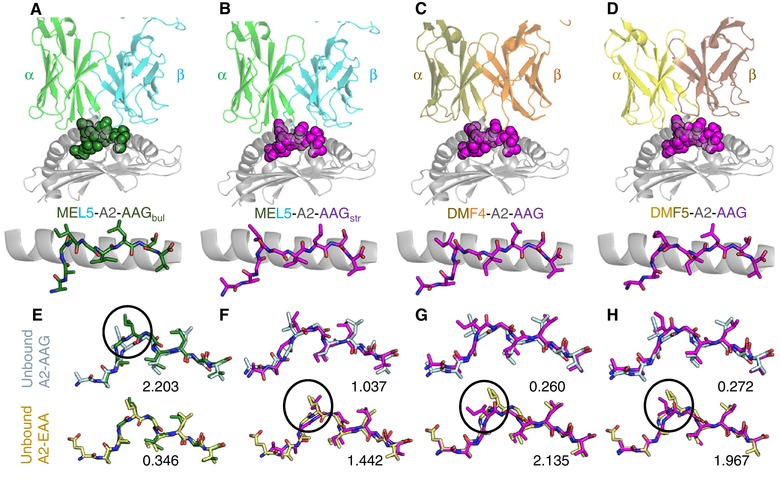
MEL5 captures A2‐AAG in a unique conformation compared to other reported TCRs. (A and B) Upper panel shows the MEL5 TCR (α‐chain green and β‐cyan cartoon) bound to HLA‐A*02:01 (grey cartoon) in complex with (A) AAGIGILTV presented in the “bulged” conformation (A2‐AAG_bul_) colored in green spheres and (B) AAGIGILTV presented in the “stretched” (A2‐AAG_str_) conformation colored in purple spheres. The lower panel shows the side view of the peptide conformation in sticks (colors as in the upper panel). (C) Upper panel shows the DMF4 TCR (sand and orange cartoon) bound to HLA‐A*02:01 (grey cartoon) in complex with AAGIGILTV (purple spheres). The lower panel shows the side view of the peptide conformation in sticks (colors as in the upper panel). (D) Upper panel shows the DMF5 TCR (yellow and brown cartoon) bound to HLA‐A*02:01 (grey cartoon) in complex with AAGIGILTV (purple spheres). The lower panel shows the side view of the peptide conformation in sticks (colors as in the upper panel). (E–H) Superpositions of A2‐AAG in complex with TCR (colored as in A–D, shown in sticks) with unbound A2‐AAG (upper panel, light blue sticks) or unbound A2‐EAA (lower panel, yellow sticks). The number below each peptide represents the root mean square deviation for each superposition. The circles show the main region where differences in peptide conformation are apparent between the TCR‐A2‐AAG complex and unbound pMHCs. (E) MEL5‐A2‐AAG_bul_, (F) MEL5‐A2‐AAG_str_, (G) DMF4‐A2‐AAG, and (H) DMF5‐A2‐AAG. These data were generated from a single data set derived from X‐ray crystallographic analysis.

The two different conformations of the AAG peptide (Fig. 4A and B) were similar to that of the AAG nonapeptide and EAA decapeptide observed in other studies. In the A2‐AAG_str_ conformation, the AAG peptide closely resembled that observed for the unbound pMHC, and the conformation present in the co‐complex structures with the DMF4 and DMF5 TCRs (Fig. 4C and D). In the A2‐AAG_bul_ form, the AAG peptide adopted a conformation similar to the A2‐EAA decapeptide that is better recognized by all other TCRs currently described. Structural alignment analysis supported this observation, demonstrating a closer match between A2‐AAG_bul_ and A2‐EAA by root mean square deviation (r.m.s.d. = 0.346 Å) than A2‐AAG_bul_ and unbound A2‐AAG (r.m.s.d. = 2.203 Å; Fig. 4E). A2‐AAG_str_ and A2‐AAG in complex with DMF4 and DMF5 all aligned more closely with unbound A2‐AAG (average r.m.s.d. = 0.523 Å) than A2‐EAA (average r.m.s.d. = 1.848 Å; Fig. 4F–H). This unique recognition mechanism likely contributes to the stronger comparative affinity between MEL5 and A2‐AAG compared to other TCRs reported.

### MEL5‐like TCRs utilize an A2‐EAA‐like peptide binding mode when interacting with A2‐AAG

**Figure 5:**
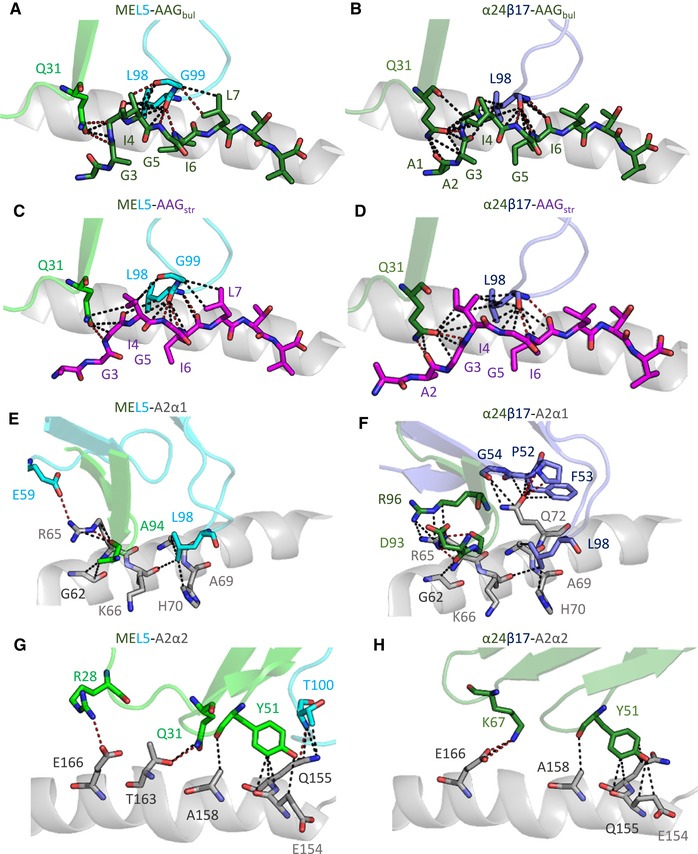
MEL5‐like TCRs can utilize an A2‐EAA‐like peptide binding mode when interacting with A2‐AAG. (A–D) TCR interactions with the AAG peptide. (E–H) TCR interactions with HLA‐A*02:01. In all panels, MEL5 is colored in green and cyan cartoon, α24β17 is colored in dark green and dark purple cartoon, HLA‐A*02:01 is colored in grey cartoon, AAG_bul_ is colored in green sticks, and AAG_str_ is colored in purple sticks. Black dotted lines represent vdW contacts (≤4 Å) and red dotted lines represent H‐bonds or salt bridges (≤3.4 Å). (A) Main interactions between MEL5 and AAG_bul_. (B) Main interactions between MEL5 and AAG_str_. (C) Main interactions between α24β17 and AAG_bul_. (D) Main interactions between α24β17 and AAG_str_. (E) Main interactions between MEL5 and HLA‐A*02:01 α1 domains. (F) Main interactions between α24β17 and HLA‐A*02:01 α1 domains. (G) Main interactions between MEL5 and HLA‐A*02:01 α2 domains. (H) Main interactions between α24β17 and HLA‐A*02:01 α2 domains. These data were generated from a single data set derived from X‐ray crystallographic analysis.

Previous co‐complex structures of the DMF4 and DMF5 TCRs have demonstrated that A2‐AAG is recognized in a “stretched” conformation compared to the “bulged” presentation mode of the decapeptide 24. This is perhaps not surprising as analysis of the peptide–MHC interaction showed that there were more contacts between HLA‐A*02:01 and AAG_str_ (122 contacts) than for AAG_bul_ (107 contacts). This was mainly due to the unoccupied A pocket and thus the loss of contact between the MHC helices and Ala1 for AAG_bul_. Thus, A2‐AAG_str_ is probably more stably presented than A2‐AAG_bul_ (consistent with the unbound A2‐AAG structure 25, 35). DMF4 underwent the largest adjustment when binding to A2‐AAG (consistent with the weaker binding affinity of *K*_D_ = 170 μM), with the TCR orienting differently compared to the interaction with the position 2 substituted heteroclitic decapeptide, HLA‐A2‐E**L**AGIGILTV (A2‐ELA). DMF5 bound to A2‐AAG in the same conformation as for A2‐ELA, probably contributing to the stronger comparative binding affinity (*K*_D_ = 40 μM), but still made substantial adjustments to peptide contacts. Both DMF4 and DMF5 had three less hydrogen bonds with the AAG peptide compared to A2‐ELA. In contrast, MEL5 bound to A2‐AAG in a virtually identical conformation to A2‐ELA and A2‐EAA and maintained a very similar peptide interface, only losing substantial contacts with Glu1 (not present in the nonapeptide) (Supporting Information Table S2). MEL5 and α24β17 interacted with A2‐AAG_bul_ and A2‐AAG_str_ in a similar fashion, both focusing on the solvent exposed “GIGI” motif in the center of the peptide and making four hydrogen bonds and multiple van der Waals (vdWs) contacts through TCR residues αGln31 and βLeu98 (Fig. 5A–D). This was reflected by a relatively small r.m.s.d. when aligning the AAG_bul_ peptide bound to MEL5 or α24β17 TCRs (r.m.s.d. = 0.335), or the AAG_str_ peptide bound to MEL5 or α24β17 TCR (r.m.s.d. = 0.538). The main differences in peptide interactions were observed at the N‐terminus of the peptide forms, in which additional contacts were formed between MEL5 αGln31 and Ala2, and α24β17 αGln31 and Ala1. Despite the similarity in number of contacts between the two forms of the peptide with MEL5, both BSA and surface complementarity were higher for MEL5‐A2‐AAG_bul_ (655 Å^2^ and 0.634) compared to MEL5‐A2‐AAG_str_ (545 Å^2^ and 0.579), suggesting that A2‐AAG_bul_ could be a more favorable conformation for MEL5 binding. Interestingly, MEL5 bound to A2‐AAG with a slightly stronger affinity than to A2‐ELA (Table 1), demonstrating the superior ability of MEL5 to recognize the natural MART‐1/Melan‐A nonapeptide compared to the heteroclitic decapeptide that has been used in vaccine trials 16, 17, 18, 19, 20, 21. This difference in affinity was consistent with the extra hydrogen bond formed between MEL5 βLeu98 and A2‐AAG Ile6, compared to the MEL5‐A2‐ELA complex 27.

An identical network of TCR–MHC contacts supported the interactions with the A2‐AAG_bul_ and A2‐AAG_str_ versions of the peptide for both MEL5 and α24β17 TCRs. Guided by interactions with all three of the restriction triad residues (Arg65, Ala69, and Gln155), MEL5 formed an additional hydrogen bond and salt bridge with MHC residues Thr163 and Glu166, respectively. The α24β17 TCR also contacted all three restriction triad residues, but formed several new interactions, primarily with the HLA α1 helix, mediated mainly through residues mutated during the affinity maturation process (Fig. 5E–H). These interactions included an extra salt bridge between αAsp93 and αArg65, and three new hydrogen bonds between TCR residues βPro52 and βPhe53, and MHC residue Gln72 compared to the MEL5 TCR. In total, α24β17 made one additional electrostatic interaction and four additional vdWs contacts with the peptide, but three additional electrostatic interactions and 56 additional vdWs contacts with the MHC surface compared to the MEL5 TCR (Table 2). Thus, in agreement with our previously published data comparing natural and modified high affinity TCRs 29, 36, 37, the high affinity interaction between α24β17 and A2‐AAG (*K*_D_ = 26.2 nM) was mainly mediated through extra contacts directly involving mutated residues.

### Thermodynamic, NMR, and modeling analysis support an anchor residue shift during MEL5 binding

**Figure 6:**
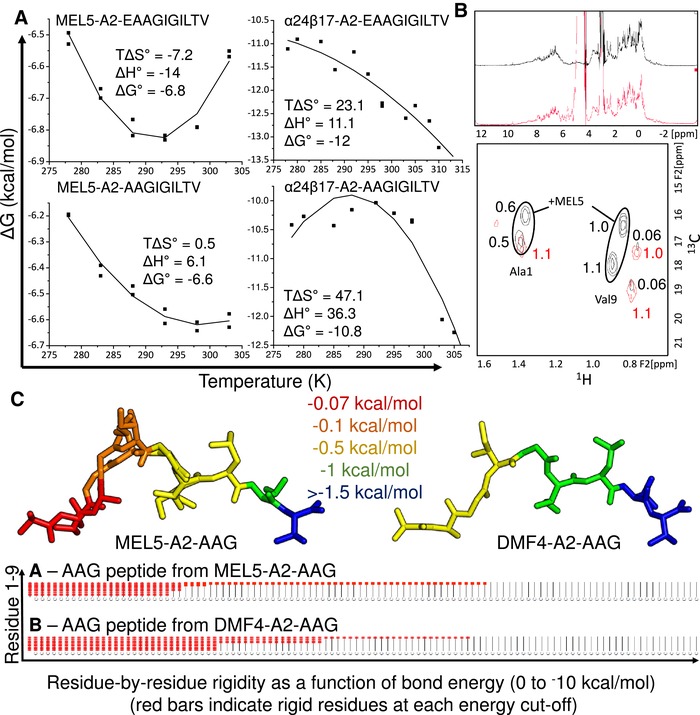
Thermodynamic, NMR, and modeling analysis support an anchor residue shift during MEL5 binding. (A) For MEL5, *K*_D_ values were measured in duplicate (separate experiments) at 5, 10, 15, 20, 25, and 30°C using equilibrium analysis by SPR. For α24β17, *K*_D_ values were measured in duplicate (separate experiments) at 5, 7, 12, 15, 19, 22, 25, 30, and 32°C using single cycle kinetic analysis by SPR; representative data from one of these experiments is plotted. From these data, the binding free energies (ΔG = RT ln *K*_D_) were plotted against temperature and the thermodynamic parameters (Δ*H*° and *T*Δ*S*°) were calculated according to the nonlinear van't Hoff equation (RT ln *K*_D_ = Δ*H*° – *T*Δ*S*° + Δ*Cp*°(*T* – *T*_0_) – *T*Δ*Cp*° ln (*T*/*T*_0_)) for MEL5‐A2‐EAA, MEL5‐A2‐AAG, α24β17‐A2‐EAA, and α24β17‐A2‐AAG. (B) Solvent‐suppressed ^1^H (top) and methyl‐region ^1^H‐^13^C HSQC NMR spectra of an equimolar mixture of A2‐A(^13^C_3_)AGIGILTV and A2‐AAGIGILTV(^15^N,^13^C_5_) in the absence (red) and presence (black) of the MEL5 TCR. Relative integrals for each HSQC peak are indicated on the spectra. These data were generated from a single data set derived from NMR analysis. (C) FIRST analysis of AAG peptide stability for the MEL5‐A2‐AAG (left) and DMF4‐A2‐AAG (right) complexes. The colors indicate the simulated energy (kcal/mol) required to destabilize different parts of the peptide with most unstable residues colored red and most stable residues colored blue. A more detailed key of the energy cutoffs is shown in the figure. Below is the stripy plot analysis, which reports the distribution of rigid clusters in the structure. The AAG peptide (full plot shown in Supporting Information Fig. S3) is shown in the context of each complex. Peptide residues 1–9 are shown from bottom to top. Red bars indicate a rigid residue. Disappearance of red bars with increased simulated bond energy is shown from left to right. These data were generated from a single data set derived from X‐ray crystallographic analysis.

Although the observations from the crystal structure were consistent with the ability of MEL5 to bind to A2‐AAG with stronger affinity than for other TCRs, we performed thermodynamic experiments to provide additional evidence for the AAG anchor residue shift upon MEL5 TCR binding. First, we analyzed changes in standard free energy (Δ*G*°), enthalpy (Δ*H*°), and entropy (Δ*S*°) for MEL5 and α24β17 interacting with A2‐AAG versus A2‐EAA (Fig. 6A). We have previously shown that the MEL5‐A2‐EAA interaction is not characterized by an anchor residue shift in the peptide, but still involves peptide side chain movement at Glu1 compared to the MEL5‐A2‐ELA complex 27, 28. This movement probably contributed toward a larger entropic penalty during binding (*T*Δ*S*° ≅ −7.2 kcal/mol) compared to MEL5‐A2‐ELA (*T*Δ*S*° ≅ 8.4 kcal/mol), that was overcome through the formation of new electrostatic interactions (in part mediated by the movement a Glu1 in the MEL5‐A2‐EAA structure). Although the standard free energies were similar for MEL5‐A2‐EAA (Δ*G*° = −6.8 kcal/mol) and MEL5‐A2‐AAG (ΔG° = −6.6 kcal/mol), MEL5 used different energetic strategies to bind to each peptide (Fig. 6A). Whereas the binding to A2‐EAA was entropically unfavorable (*T*Δ*S*° = −7.2 kcal/mol), the binding to A2‐AAG was slightly entropically favored (TΔS° = 0.49 kcal/mol). This is consistent with the extra mobility observed in the peptide in the MEL5‐A2‐AAG structure, which would result in a lower disorder to order transition during ligand engagement. The thermodynamics were also consistent with the interaction interface, with the MEL5‐A2‐EAA complex forming more bonds (12 electrostatics and 104 vdW's, compared to nine electrostatics and 75 vdW's for MEL5‐A2‐AAG) and having a more favorable enthalpic change (ΔH° = −14 kcal/mol), compared to the MEL5‐A2‐AAG complex (Δ*H*° = −6.1 kcal/mol). The thermodynamic analysis of the α24β17 TCR was consistent with these observed differences in A2‐EAA versus A2‐AAG TCR interactions, demonstrating a more favorable entropic component (*T*Δ*S*° = 23.1 kcal/mol and TΔS° = 47.1 kcal/mol) and less favorable enthalpic component (Δ*H*° = 11.1 kcal/mol and Δ*H*° = 36.3 kcal/mol) for binding to A2‐EAA and A2‐AAG, respectively. Although α24β17 is a modified high affinity TCR, these data represent the most extreme unfavorable entropic component observed for a TCR‐pMHC interaction (range observed for natural TCR‐pMHC interactions: Δ*H*° = −30 to 12 kcal/mol; *T*Δ*S*° = −24 to 8.4 kcal/mol) 27, 38, extending the range of energetic strategies that can be utilized by the TCR scaffold.

Next, we examined the conformational consequences of MEL5 binding to A2‐AAG in solution using NMR spectroscopy (Fig. 6B). The AAG peptide was labeled with ^13^C at Ala1 (which underwent the largest movement between the AAG_bul_ and AAG_str_ structures) and Val9 (which did not move upon MEL5 binding) and examined by NMR alone and in the presence of the MEL5 TCR. 1D ^1^H spectra showed that proteins were folded under both conditions. In the absence of MEL5, each labeled methyl group gave a single resonance, with approximately equal intensities to one another. On addition of MEL5, all labeled methyl groups showed chemical shift perturbations, and multiple resonances were seen for each methyl group. However, while the Val9 methyl groups each gave one major and one minor resonance (consistent with there being a small amount of A2‐AAG binary complex still present), the Ala1 methyl group gave two resonances of approximately equal intensity to one another, and half the intensity of the Val9 methyl peaks. The observation of two resonances of ≈50% intensity mirrors our findings in the crystal structure in which the AAG_bul_ and AAG_str_ forms of the peptide were both present when in complex with MEL5. This suggests that, in the presence of MEL5, the Ala1 methyl group experiences two different molecular environments while the Val9 methyl groups only experience one, consistent with the X‐ray structure.

Finally, we investigated the stability of the AAG peptide in complex with MEL5 and DMF4 using a computational approach, FIRST, as described in the methods (Supporting Information Fig. S3A and B). This analysis demonstrated that the AAG peptide was more flexible when bound to MEL5 compared to DMF4 (Fig. 6C), particularly at the N‐terminus (the portion of the peptide that shifted when binding to MEL5). The first two residues of the peptide (AA) lost rigidity at cutoffs of –0.07 kcal/mol or smaller in the MEL5 complex, compared to the DMF4 complex where the same residues remained rigid down to a cutoff of –0.27 kcal/mol. In contrast, the C‐terminal portion of the AAG peptide in both the MEL5 and DMF4 structures was still rigid at cutoffs around –1.5 kcal/mol. Thus, these analyses support our other observations demonstrating that the MEL5 TCR, but not other A2‐AAG/A2‐EAA specific TCRs, can induce N‐terminal flexibility in the AAG peptide that enhances TCR engagement.

## Discussion

Despite over a decade of research, therapies directed against the HLA‐A*02:01 restricted MART‐1/Melan‐A_26/27‐35_ peptide antigens have had limited success 39, 40. Approaches that employed a heteroclitic version of the decapeptide with the position 2 Ala substituted for Leu (E**L**AGIGILTV) probably failed because the anchor residue modification at peptide residue 2 altered the TCR recognition platform 28, 41, leading to selection of T cells that did not recognize the native antigen effectively 18, 40. Furthermore, although the majority of MART‐1/Melan‐A specific CD8^+^ T cells preferentially recognize the decapeptide rather than the nonapeptide 17, 24, the MART‐1/Melan‐A_27‐35_ nonapeptide, AAGIGILTV, has been identified as the immunodominant epitope on the surface of tumors 22. Subsequently, TCRs selected against the decapeptide have generally been found to recognize the nonapeptide poorly 24, 41, probably because the nonapeptide forms a different conformation when presented by HLA‐A*02:01 compared to the decapeptide 25, 26, adding a further explanation for the poor outcome of therapies directed against this antigen.

To shed additional light on this therapeutically important system, we focused on the MEL5 TCR that can bind to A2‐AAG with stronger affinity than any other TCR reported 23, 24. We examined the structural and thermodynamic properties of the MEL5‐A2‐AAG interaction (and used a high affinity version of the MEL5 TCR, α24β17, to validate our observations) and compared our findings to DMF4 and DMF5 recognition of MART‐1/Melan‐A_26/27‐35_. Our data revealed that recognition of A2‐AAG by MEL5, but not other published TCRs, involved a novel peptide anchor residue switch, enabling the peptide to present a bulged form, anchored with Ala1 of the peptide within the MHC B‐pocket. Our data are consistent with the recognition patterns of the heteroclitic nonapeptides, LAGIGILTV and ALGIGILTV. The LAGIGILTV peptide that forms a bulged conformation similar to A2‐EAA, (because the Ala1Leu mutation forces the peptide to anchor at position P1 in the B pocket), is recognized optimally compared to ALGIGILTV 17, which is forced into the stretched conformation 25. Thus, unlike the DMF4 and DMF5 TCRs, where the complex structures with A2‐ELA and A2‐AAG were out of alignment at Ile4 and Ile5, MEL5 could stabilize the bulged nonapeptide conformation, mimicking the A2‐ELA, A2‐EAA, and A2‐LAG peptide conformations upon TCRs recognition.

The peptide anchor residue shift in the MEL5‐A2‐AAG and α24β17‐A2‐AAG structures was further supported by the thermodynamic and NMR experiments. In both cases, thermodynamic analyses of the TCR‐A2‐AAG complexes revealed an entropically more favorable interaction than for the TCR‐A2‐EAA complexes. Further, chemical shift analysis by NMR demonstrated that, on addition of MEL5, two resonances of equal intensities were apparent for the labeled Ala1 residue (where we observed the anchor residue shift in the structure), consistent with the existence of two different conformations for this residue represented by the AAG_bul_ and AAG_str_ forms of the peptide in the crystal structure. Lastly, FIRST rigidity analysis was also consistent with our findings, demonstrating greater peptide instability at the N‐terminus of the AAG peptide when bound to MEL5 compared to the DMF4 TCR. The mechanism that drives the conformational change we observed in the AAG peptide when bound to MEL5, and not other TCRs, remains unclear. We have previously shown the importance of Gln at position 31 in the CDR1 loop of MEL5 (encoded by TRAV12‐2) for recognition of the A2‐EAA and A2‐ELA decamer peptides 27, 28. However, although DMF4 lacks this residue (encoded by TRAV35), the DMF5 TCR shares the TRAV12‐2 gene usage and uses a very similar binding mode, compared to MEL5, to engage both the AAG nonomer and EAA decamer peptides. We speculate that fine differences in the dynamics of the CDR loops of MEL5, compared to DMF4 and DMF5 TCRs, enable its unique binding mode, further demonstrating the complexity of the mechanisms that underpin TCR recognition of pMHC.

These findings have implications for the mechanisms that control the high degree of T‐cell cross‐reactivity recently reported 7, 8, 9, 10, 11, 12, 13. One key idea, which has arisen through observations made through molecular experimentation using X‐ray crystallography and other techniques, is that the TCR–pMHC interaction is highly flexible 3, 42, 43. Indeed, studies to date have revealed three main mechanisms of conformational flexibility at the TCR‐pMHC interface that likely play a key role in T‐cell antigen recognition. First, it has been shown that the TCR CDR loops can flex upon binding 3. The types of loop shift can be placed into three major classes: (i) loop remodeling facilitated by multiple φ/ψ bond angle changes; (ii) hinge‐bending motions; and (iii) rigid‐body shifts 3. Second, movements of the MHC α‐helices have been described in which the α‐helices flanking the peptide‐binding groove can shift upon TCR binding. This has been observed in the recognition of H‐2K^b^‐RGYVYQGL by the BM3.3 TCR 44, 45 and HLA‐DR2a‐VHFFKNIVTPRTPG by the 3A6 TCR 46. In both cases, rigid‐body shifts of ≈1 Å were distributed across both the α1 and α2 helices 3. More radical HLA‐A*02:01 α2‐helix conformational changes have also been observed upon the recognition of A2‐MLWGYLQYV by the A6 TCR in which a conformational “switch” occurred at the hinge of the short and long helical elements that altered the pivot of the short arm and extended the long arm of the α2‐helix 47. Third, there have been reports that TCR binding can induce conformational changes in the solvent exposed central peptide residues, mediating TCR docking and T‐cell recognition 48. Small backbone shifts have been observed upon recognition of the A2‐LLFGYPVYV and A2‐SLLMWITQC peptides presented by HLA‐A*02:01 49, 50, 51, 52. More dramatic peptide movements have been reported for binding of the ELS4 TCR to HLA‐B*35:01‐EPLPQGQLTAY, with a large shift of 5 Å occurring in the center of the peptide 5. Also, a recent study demonstrated that two different TCRs, recognizing the same HLA‐B*07:02 restricted NY‐ESO‐1 peptide, could stabilize the peptide in very different conformations 53. Our study adds to the story of flexibility during T‐cell antigen recognition, through a TCR induced shift in peptide primary anchor residue usage.

In summary, this study sheds new light on the mechanisms that control T‐cell recognition of an important tumor antigen and provides some explanations as to why therapeutic approaches have not been successful in this system. We demonstrate that the MEL5 TCR, unlike other reported TCRs, was able to induce an anchor residue switch in the dominant AAGIGILTV nonapeptide, enabling recognition of the peptide in the optimal EAAGIGILTV‐like conformation. This TCR recognition mechanism likely contributes to the ability of MEL5 to bind to A2‐AAG with stronger affinity than other reported TCRs 23, 24. These data, combined with our previous findings 23, suggest that future therapeutic approaches should focus on the selection of MEL5‐like TCRs for optimal tumor recognition. Finally, we reveal an extended mechanism of flexibility at the TCR–pMHC interface that contributes to our understanding of the molecular rules that govern T‐cell antigen recognition.

## Materials and methods

### Generation of MEL5 TCR and α24β17 TCR and cell culture

The MEL5 TCR was derived from the MEL5 CD8^+^ T‐cell clone specific for HLA‐A*02:01 MART‐1_26‐35_ and is previously described 27, 54. The α24β17 TCR, a high‐affinity version of the MEL5 TCR, was generated by phage display as described previously 55. Melanoma cell lines Mel562 (HLA‐A2^+^), Mel624 (HLA‐A2^+^), and SK‐MEL‐28 (HLA‐A2^neg^), breast adenocarcinoma MDA‐MB‐231(HLA‐A2^+^), and lymphoblast cell line T2 (HLA‐A2^+^) were obtained from laboratory stocks. All cell lines were cultured in RPMI 1640 supplemented with 10% fetal bovine serum (FBS), penicillin/streptomycin, and l‐glutamine (all from Gibco, Paisley, UK), and routinely tested for Mycoplasma infection, and found negative (MycoAlert™, Mycoplasma Detection Kit, Lonza).

### Generation of TCR‐transduced T cells

TCR transduced T‐cells were generated as described previously 56. In brief, the following TCRs were codon optimized, synthesized (GeneArt™, Thermo Fisher Scientific) and cloned into a third generation lentiviral vector pELNS (kindly provided by James Riley, University of Pennsylvania, PA): MEL5 27, MEL187.c5 41, and DMF4 57. TCR‐α and TCR‐β chains, as well as rat CD2 marker gene were separated with self‐cleaving 2A sequences 58 to ensure their stoichiometric expression. Lentiviral particles were generated in HEK293T cell line by calcium phosphate transfection of TCR‐encoding pELNS plasmid together with packaging plasmids pRSV‐Rev, pMDLg/pRRE, and envelope plasmid pMD2.G. Prior to T‐cell transduction, lentiviral particles were concentrated 100× by ultracentrifugation.

PBMCs were isolated from healthy donor buffy coats (Welsh Blood Service, Pontyclun, UK) in accordance with local ethical approval and national legislation. PBMC isolation was performed by Lymphoprep (Axis Shield, Olso, Norway) density gradient centrifugation using SepMate tubes (STEMCELL Technologies, Vancouver, BC, Canada). CD8^+^ T cells were isolated from three healthy donors’ PBMC using magnetic CD8 MicroBeads (Miltenyi Biotec) according to manufacturer's recommendations. T cells were then activated with CD3/CD28 beads (Dynabeads® Human T‐Activator, Thermo Fisher Scientific) at three beads to one T‐cell ratio for 24 h prior to lentiviral transduction. Concentrated lentiviral particles were then added to T cells in presence of 5 μg/mL polybrene (Santa Cruz Biotech). T cells were cultured in T‐cell media, composed of RPMI 1640 supplemented with 10% FBS, penicillin/streptomycin, l‐glutamine, 10 mM HEPES, 1 mM sodium pyruvate, 1× nonessential amino acids, 25 ng/mL IL‐15 (Peprotech, Rocky Hill, NJ), and 200 IU IL‐2 (Prometheus, San Diego, CA). On day 10 post transduction, T cells that had taken up lentivirus were purified using anti‐rat CD2 PE‐conjugated antibody (OX‐34, Biolegend) followed by anti‐PE MicroBeads (Miltenyi Biotec). On day 14, T cells were expanded in T‐cell media containing 20 IU/mL IL‐2 and in presence of allogeneic irradiated feeder cells from three donors and 1 μg/mL phytohemagglutinin as described previously 59. From day 14 post expansion, T cells were used for functional experiments without cryopreservation. In all functional assays, TCR‐transduced T cells were >90% CD8^+^ rat CD2^+^. T‐cell viability was routinely measured by Trypan blue exclusion counting prior to functional experiments. Cell viability was never below 90%.

### Functional T‐cell assays

T cells were rested for 24 h prior to functional assays in RPMI 1640 medium supplemented with 5% FBS. All functional assays were performed in this medium. Flow cytometry: 50 000 T cells were co‐incubated with 50 000 cancer cells in presence of 30 μM TNF processing inhibitor (TAPI‐0, Sigma–Aldrich) and anti‐TNF antibody (cA2, Miltenyi Biotec) for 5 h. Following co‐incubation, cells were washed with PBS and stained with LIVE/DEAD Violet Fixable Dead Cell stain (Life Technologies), as well as anti CD3‐PerCP (BW264/56, Miltenyi Biotec), anti‐CD8 (BW135/80, Miltenyi Biotec), and anti‐rat CD2 antibodies (OX‐34, Biolegend). The cells were gated on lymphocytes based on forward and side scatter, followed by sequential gating to include only viable CD8^+^ cells (and rCD2^+^, in case of TCR transduced cells). A minimum of 5000 live events were acquired on FACS Canto II (BD Biosciences), and the analysis was performed using FlowJo software (TreeStar Inc, Ashland, OR) and GraphPad Prism.

#### Enzyme‐linked immunosorbent assay

A total of 30 000 T‐cells were incubated with 30 000 T2 cells in presence of titrated concentrations of ELAGIGILTV, EAAGIGILTV, and AAGIGILTV peptides for 18 h. T cells incubated alone or with 10 μg/mL phytohemagglutinin were used as negative and positive controls, respectively, to determine the maximum response. Following co‐incubation, concentration of secreted MIP‐1β and TNF were measured using Human MIPβ or TNF DuoSet kit (R&D Systems), according to manufacturer's recommendations. The experiment was performed in duplicate. Data analysis was performed in GraphPad Prism.

### Cloning, expression, and refolding of proteins

The TCR‐α and TCR‐β chains as well as the MHC class I α‐chains (tagged and not tagged with a biotinylation sequence) and β2m sequences were cloned into the pGMT7 expression vector under the control of the T7 promoter using BamH1 and EcoR1 restriction sites as described previously 60, 61, 62. All sequences were confirmed by automated DNA sequencing.

The TCR‐α and TCR‐β chains, the HLA‐A*02:01 α chains, and β2m were expressed separately, without posttranslational modification, as insoluble inclusion bodies in competent Rosetta DE3 *Escherichia coli* cells (Merk) using 1 mM IPTG as described previously 60, 61, 62.

For a 1 L TCR refold, 30 mg TCR‐α chain was incubated at 37°C for 30 min with 10 mM DTT and added to cold refold buffer (50 mM TRIS pH 8.1, 2 mM EDTA, 2.5 M urea, 6 mM cysteamine hydrochloride, and 4 mM cystamine). After 30 min, 30 mg TCR‐β chain, also incubated at 37°C for 30 min with 10 mM DTT, was added. For a 1 L pMHC‐I refold, 30 mg HLA‐A*02:01 α‐chain was mixed with 30 mg β2m and 4 mg of peptide at 37°C for 30 min with 10 mM DTT. This mixture was then added to cold refold buffer (50 mM TRIS pH 8.1, 2 mM EDTA, 400 mM l‐arginine, 6 mM cysteamine hydrochloride, and 4 mM cystamine). TCR and pMHC‐I refolds were mixed at 4°C for >1 h and dialyzed against 10 mM TRIS pH 8.1 until the conductivity of the refolds was <2 mS/cm. All the refolds were then filtered, ready for purification steps.

Refolded proteins were purified initially by ion exchange using a Poros50HQ^TM^ column (Thermo Fisher Scientific Inc, MA, USA) and finally gel filtered into crystallization buffer (10 mM TRIS pH 8.1 and 10 mM NaCl) or BIAcore buffer (10 mM HEPES pH 7.4, 150 mM NaCl, 3 mM EDTA, and 0.005% (v/v) surfactant P20) using a Superdex200HR^TM^ column (GE Healthcare, Buckinghamshire, UK). Protein quality, either under nonreducing or reducing conditions, was analyzed by Coomassie‐stained SDS‐PAGE.

### Protein crystallization and structure determination

Crystals were grown at 18°C by vapor diffusion via the sitting drop technique. All crystallization‐screening and optimization experiments were completed with an Art‐Robbins Phoenix dispensing robot (Alpha Biotech Ltd, U.K.). Two hundred nanoliters of 10–15 mg/mL TCR–pMHC complex mixed at a 1:1 molar ratio was added to 200 nL of reservoir solution. Intelli‐plates were then sealed and incubated at room temperature in a crystallization incubator (18°C) (RuMed, Rubarth Apperate GmbH, Germany) and analyzed for crystal formation crystal formation using the Rock Imager 2 (Formulatrix, Bedford, MA USA). Crystals selected for further analysis were cryoprotected with ethylene glycol to 25% and then flash cooled in liquid nitrogen in Litho loops (Molecular Dimensions, UK). For MEL5‐A2‐AAG, optimal crystals were obtained in TOPS 63 with 0.1 M HEPES pH 7.5, 25% PEG 4000, and 15% glycerol. For α24β17‐A2‐AAG, optimal crystals were obtained in TOPS 63 with 0.1 M HEPES pH 7.0, 20% PEG 4000, and 15% glycerol.

Diffraction data were collected at several different beamlines at the Diamond Light Source (Oxford, UK) using a Pilatus 2M detector, a QADSC detector or a Rayonix detector. Using a rotation method, 400 frames were recorded each covering 0.5° of rotation. Reflection intensities were estimated with the XIA2 package 64, 65 and the data were scaled, reduced, and analyzed with SCALA and the CCP4 package 66. The TCR‐pMHC complex structures were solved with molecular replacement using PHASER 67. The model sequences were adjusted with COOT 68 and the models refined with REFMAC5. Accession code MEL5‐A2‐AAG: PDB 6EQA and α24β17‐A2‐AAG: PDB 6EQB.

### pMHC biotinylation and surface plasmon resonance analysis

Biotinylated pMHCs were prepared as described previously 69. Binding analysis was performed using a BIAcore T100™ or a BIAcore® 3000 (GE Healthcare, Buckinghamshire, UK) equipped with a CM5 sensor chip. Briefly, CM5 chip coupling solutions containing 100 μL of 100 mM NHS and 100 μL of 400 mM EDC were used to activate the chip prior to streptavidin binding. Approximately 5000 response units (RU) of streptavidin (110 μL of 200 μg/mL in 10 mM acetate pH 4.5) was covalently linked to the chip surface in all four flow cells and 100 μL of 1 M ethanolamine hydrochloride was used to deactivate any remaining reactive groups. Approximately 200–500 RU of pMHC was attached to the CM5 sensor chip at a slow flow rate of 10 μL/min to ensure uniform distribution on the chip surface. Combined with the small amount of pMHC bound to the chip surface, this reduced the likelihood of off‐rate‐limiting mass transfer effects. HLA‐B*81:01‐TPQDLNTML‐Gag_180‐188_ 70 and HLA‐B*51:01‐TAFTIPSI‐HIV‐RT_128‐135_ 71 were used as negative controls. MEL5 was purified and concentrated to ≈120 μM and α24β17 was purified and concentrated to ≈0.5 μM on the same day of surface plasmon resonance analysis to reduce the likelihood of TCR aggregation affecting the results.

For equilibrium and kinetic analysis with MEL5, 10 serial dilutions (1/2) were prepared in triplicate for each sample and injected over the relevant sensor chips at 25°C. MEL5 was injected over the chip surface using kinetic injections at a flow rate of 45 μL/min. For the thermodynamics experiments, this method was repeated at the following temperatures: 5, 10, 15, 20, 25, and 30°C. Results were analyzed using BIAevaluation 3.1 (GE Healthcare, Buckinghamshire, UK), Excel and Origin 6.0 software. The equilibrium binding constant (*K*_D_) values were calculated assuming a 1:1 interaction (A+B↔AB) by plotting specific equilibrium‐binding responses against protein concentrations followed by nonlinear least squares fitting of the Langmuir binding equation$\;\mathrm{AB}\;=\frac{B\times ABmax}{K_{D}+B}\;$. The thermodynamic parameters were calculated using the nonlinear van't Hoff equation (RT ln *K*_D_ = Δ*H*° – *T*Δ*S*° + Δ*Cp*°(*T* – *T*_0_) – *T*Δ*C*p° ln (*T*/*T*_0_)) with *T*_0_ = 298 K.

For surface plasmon resonance analysis with α24β17, the single‐cycle kinetics method 72 was used. Five serial dilutions (1/3) were prepared in duplicate for each sample and the TCR was injected at 25°C at a flow rate of 45 μL/min for 200 s. The dissociation time between each injection was 120 s and the dissociation time after the last injection was 3600 s. Association constant (*k*_on_), dissociation constant (*k*_off_), and affinity constant (*K*_D_) were estimated by global fitting of the data using BIAevaluation 3.1 software. For the thermodynamics experiments, this method was repeated at the following temperatures: 5, 7, 12, 15, 19, 22, 25, 30, and 32°C. The thermodynamic parameters were calculated using the nonlinear van't Hoff equation with Origin 6.0 software.

### NMR experiments

Solvent‐suppressed ^1^H spectra (pulse program zgesgp) and ^1^H‐^13^C HSQC spectra (pulse program hsqcetgpsisp2; ^13^C spectral window of 12.5–22.5 ppm) were acquired on a Bruker AVANCE III 600 MHz (^1^H) NMR spectrometer equipped with a QCI‐P cryoprobe at room temperature. HLA‐A*02:01 was refolded as above using a 1:1 mixture of A(^13^C_3_)AGIGILTV and AAGIGILTV(^15^N,^13^C_5_). Half of this solution was added to MEL5 TCR solution to form the ternary complex, while half was added to an equivalent volume of buffer to maintain the binary complex. The MEL5 TCR and A2‐AAG proteins were used at 1 mg/mL in PBS. Resonance assignments were confirmed using a 2D (HC)CH‐TOCSY experiment.

### Rigidity analysis using FIRST software

Peptide rigidity was simulated using FIRST rigidity analysis 73 to identify hydrophobic tethers between residues based on the proximity of nonpolar heavy atom species, essentially aliphatic or aromatic carbons. Hydrogen bonds were also identified based on the proximity of polar H and acceptor atoms (O,N), and rated by strength based on the donor–hydrogen–acceptor geometry, on an energy scale from 0 to –10 kcal/mol. A rigidity analysis of the all‐atom input structure was carried out in FIRST using the “pebble game” algorithm 74, 75, which matches degrees of freedom against bonding constraints in the molecular framework of the protein. Bonding constraints include covalent, hydrophobic and polar (hydrogen bond and salt bridge) interactions. As the strength of the polar interactions can be gauged from their geometry, the results of the analysis depend on an “energy cutoff,” which selects the set of polar interactions to include in the constraint network 73. Comparison of the cutoffs at which different features of a protein complex become flexible thus provides information on the relative rigidity and stability of these features. We report cutoff values to a precision of 0.01 kcal/mol.

## Conflict of interest

M.S., N.L., B.K.J., and D.K.C. are employees of Immunocore LTD. The rest of the authors declare no commercial or financial conflict of interest.

## Author contributions

F.M., P.J.R., M.L., C.J.H., A.F., A.B., A.J.S., J.R.H., S.A.W., A.G., J.J.M., M.S., Y.L., B.K.J., E.J.L., D.K.S., and A.K.S. performed and/or directed experiments, analyzed data, and critiqued the manuscript. D.K.C. and A.K.S. conceived, funded, and directed the project. D.K.C., M.L., P.J.R., and A.K.S. wrote the manuscript.

**Abbreviations**
BSA: buried surface area
FBS: fetal bovine serum
MART‐1: melanoma antigen recognized by T‐cells‐1
Melan‐A: melanoma antigen‐A
pMHC: peptide–MHC
vdWs: van der Waals

## Supporting information

Supporting Information
