# Supplementary material for: TCR‐induced alteration of primary MHC peptide anchor residue
Source: Eur J Immunol. 2019 May 27;49(7):1052–66. doi: 10.1002/eji.201948085 (PMC6618058; doi:10.1002/eji.201948085)
Supplement: Supplementary file 1 — Supporting Information [file EJI-49-1052-s001.pdf]

European Journal of Immunology

Supporting Information
for
DOI 10.1002/eji.201948085

Florian Madura, Pierre J. Rizkallah, Mateusz Legut, Christopher J. Holland, Anna Fuller, Anna Bulek, Andrea J. Schauenburg, Andrew Trimby, Jade R. Hopkins, Stephen A. Wells, Andrew Godkin, John J. Miles, Malkit Sami, Yi Li, Nathaniel Liddy, Bent K. Jakobsen, E. Joel Loveridge, David K. Cole and Andrew K. Sewell

TCR-induced alteration of primary MHC peptide anchor residue

© 2019 The Authors. *European Journal of Immunology* published by WILEY-VCH Verlag GmbH & Co. KGaA, Weinheim.

Supporting Information

TCR-induced alteration of primary MHC peptide anchor residue

Florian Madura[1*], Pierre J. Rizkallah[1*], Mateusz Legut[1*], Christopher J. Holland[1,4], Anna Fuller[1], Anna Bulek[1], Andrea J. Schauenburg[1], Andrew Trimby[1], Jade R. Hopkins[1], Stephen A. Wells[2], Andrew Godkin[1], John J. Miles[1,3], Malkit Sami[4], Yi Li[4], Nathaniel Liddy[4], Bent K. Jakobsen[4], E. Joel Loveridge[5,6], David K. Cole[1,4*] and Andrew K. Sewell[1,7*]

[1]Cardiff University School of Medicine, Heath Park, Cardiff, CF14 4XN, UK.

[2]Department of Chemical Engineering, University of Bath, Bath, UK.

[3]Centre for Biodiscovery and Molecular Development of Therapeutics, Australian Institute of Tropical Health and Medicine, James Cook University, Cairns, QLD, Australia.

[4]Immunocore Ltd., Milton Park, Abingdon, UK.

[5]School of Chemistry, Cardiff University, Park Place, Cardiff, CF10 3AT, UK.

[6]Department of Chemistry, Swansea University, Singleton Park, Swansea, SA2 8PP, UK.

[7]Systems Immunity Research Institute, Cardiff University, UK.

[*]These authors contributed equally to this study.

Correspondence: Dr David Cole, E-mail: david.cole@immunocore.com. Tel: +441235 776256 and Professor Andrew Sewell, E-mail: sewellak@cardiff.ac.uk. Tel: +442920 687055.

1 Supplementary Tables

1.1 Supplementary Table S1. Data collection and refinement statistics (molecular replacement).

	MEL5-A2-AAG	α24β17-A2-AAG
**Data collection**		
PDB	6EQA	6EQB
Space Group	$P4_1$	$P4_1$
Unit Cell parameters (Å)	a=121.40, b=121.40, c=82.32	a=120.77, b=120.77, c=82.25
Radiation Source	DIAMOND I04-1	DIAMOND I03
Wavelength (Å)	0.9173	0.9363
Resolution (Å)	3.16	2.81
Unique reflections	20733	29094
Completeness (%)	100	100
Multiplicity	6.6	8.0
I/Sigma(I)	13.4	9.9
Rmerge	0.119	0.146
**Refinement**		
No reflections used	19653	27589
No reflections in Rfree set	1062	1478
Rcryst (no cutoff) (%)	18.6	20.0
Rfree (%)	26.3	25.7
**RMSD from ideal geometry (target values in parenthesis)**		
Bond lengths (Å)	0.013 (0.021)	0.016 (0.021)
Bond Angles (°)	1.431 (1.936)	1.597 (1.937)
Wilson B-factor (Å^2)	58.1	61.0
Overall ESU based on Maximum Likelihood (Å^2)	46.2	29.72

One crystal was used for data collection.

1.2 Supplementary Table S2: MEL5/α24β17-A2-AAG. 3.4Å cut-off for electrostatics and 4Å cut-off for vdW.

Table S2A	MEL5	Electrostatics (≤3.4Å)	vdW (≤4Å)
**AAG$_{bul}$** Ala2	αGln31		1
$Gly3^{N}$	$αGln31^{Oε1}$	1 HB	4
Gly3	βLeu98		2
Ile4	βLeu98		4
Ile4	βGly99		4
$Gly5^{N}$	$βLeu98^{O}$	1 HB	2
Ile6	βGly97		2
$Ile6^{N/O}$	$βLeu98^{O/N}$	2 HBs	5
Leu7	βGly99		2
Thr8	βThr96		1
**AAG$_{str}$** $Gly3^{N}$	$αGln31^{Oε1}$	1 HB	3
Gly3	βLeu98		3
Ile4	βLeu98		4
Ile4	βGly99		2
Ile4	αGln31		1
$Gly5^{N}$	$βLeu98^{O}$	1 HB	2
Ile6	βGly97		2
$Ile6^{N/O}$	$βLeu98^{O/N}$	2 HBs	5
Leu7	βGly99		2
Thr8	βThr96		2
**A2** Gly62	αAla94		3
$Arg65^{Nε}$	$αAla94^{O}$	1 HB	6
Arg65	αGly95		1
Arg65	αLys96		4
Arg65	βTyr49		5
$Arg65^{NH1}$	$βGlu59^{Oε1}$	1 HB	
Lys66	βLeu98		1
Ala69	βLeu98		2
His70	βLeu98		1
Gln72	βVal51		2
Thr73	βGly97		2
Val76	βAsn30		3
Glu154	αTyr51		3
Gln155	αTyr51		5
Gln155	βGly99		2
$Gln155^{Oε1}$	$βThr100^{N}$	1 HB	4
Ala158	αTyr51		1
Tyr159	αGln31		1
Thr163	αGly29		1
$Thr163^{Oγ1}$	$αGln31^{Nε2}$	1 HB	1
$Glu166^{Oε2}$	$αArg28^{NH2}$	1 SB	

HB = H-bond, SB = salt bridge, vdW = van der Waals contact

Table S2B		α24β17	Electrostatics (≤3.4Å)	vdW (≤4Å)
**AAG_{bul}**	Ala1^{O}	αGln31$^{N\varepsilon 2}$	1 HB	1
	Ala2	αGln31		4
	Ala2	βLeu98		1
	Gly3^{N}	αGln31$^{O\varepsilon 1}$	1 HB	5
	Gly3	βLeu98		3
	Ile4	αGln31		2
	Ile4	βLeu98		2
	Gly5^{N}	βLeu98^{O}	1 HB	3
	Ile6	βGly97		1
	Ile6$^{N/O}$	βLeu98$^{O/N}$	2 HB	8
	Thr8	βThr96		2
**AAG_{str}**	Ala2^{O}	αGln31$^{N\varepsilon 2}$	1 HB	2
	Gly3	αGln31		3
	Ile4^{N}	αGln31$^{O\varepsilon 1}$	1 HB	3
	Ile4	αAsn92		1
	Ile4	βLeu98		5
	Gly5^{N}	βLeu98^{O}	1 HB	2
	Ile6	βGly97		2
	Ile6$^{N/O}$	βLeu98$^{O/N}$	2 HB	8
	Thr8	βThr96		2
**A2**	Glu58	αPhe27		3
	Gly62	αAsp93		1
	Arg65^{NH2}	αAsp93$^{O/O\delta 1}$	1 HB, 1 SB	4
	Arg65$^{N\varepsilon}$	αGly94^{O}	1 HB	5
	Arg65	αArg96		10
	Arg65	βIle56		1
	Arg65	βSer57		3
	Lys66	αGln31		1
	Lys66	αGly94		2
	Lys66	βLeu98		1
	Lys68	βIle56		1
	Ala69	βTyr49		1
	Ala69	βIle56		1
	Ala69	βLeu98		2
	His70	βLeu98		1
	Gln72	βGly51		3
	Gln72$^{O\varepsilon 1}$	βPro52^{N}	1 HB	4
	Gln72$^{O\varepsilon 1}$	βPhe53^{N}	1 HB	7
	Gln72$^{N\varepsilon 2}$	βGly54^{O}	1 HB	3
	Gln72	βIle56		2
	Arg75	βPhe53		21
	Val76	βAsn30		2
	Val76	βPhe53		2
	Glu154	αTyr51		4
	Gln155	αTyr51		5
	Gln155	βGly99		1
	Gln155	βMet100		2
	Ala158	αTyr51		2
	Tyr159	αGln31		4
	Glu166$^{N\varepsilon 1/N\varepsilon 2}$	αLys67$^{N\zeta}$	2 HB	1
	Trp167	αGly29		2
	Arg170	αLeu28		2

Mutated residues highlighted in red.

2 Supplementary Figures

2.1 Supplementary Figure S1. Functional assessment of the response towards HLA-A*02:01/MART-1 epitopes mediated by MEL5, MEL187.c5 and DMF4 TCRs

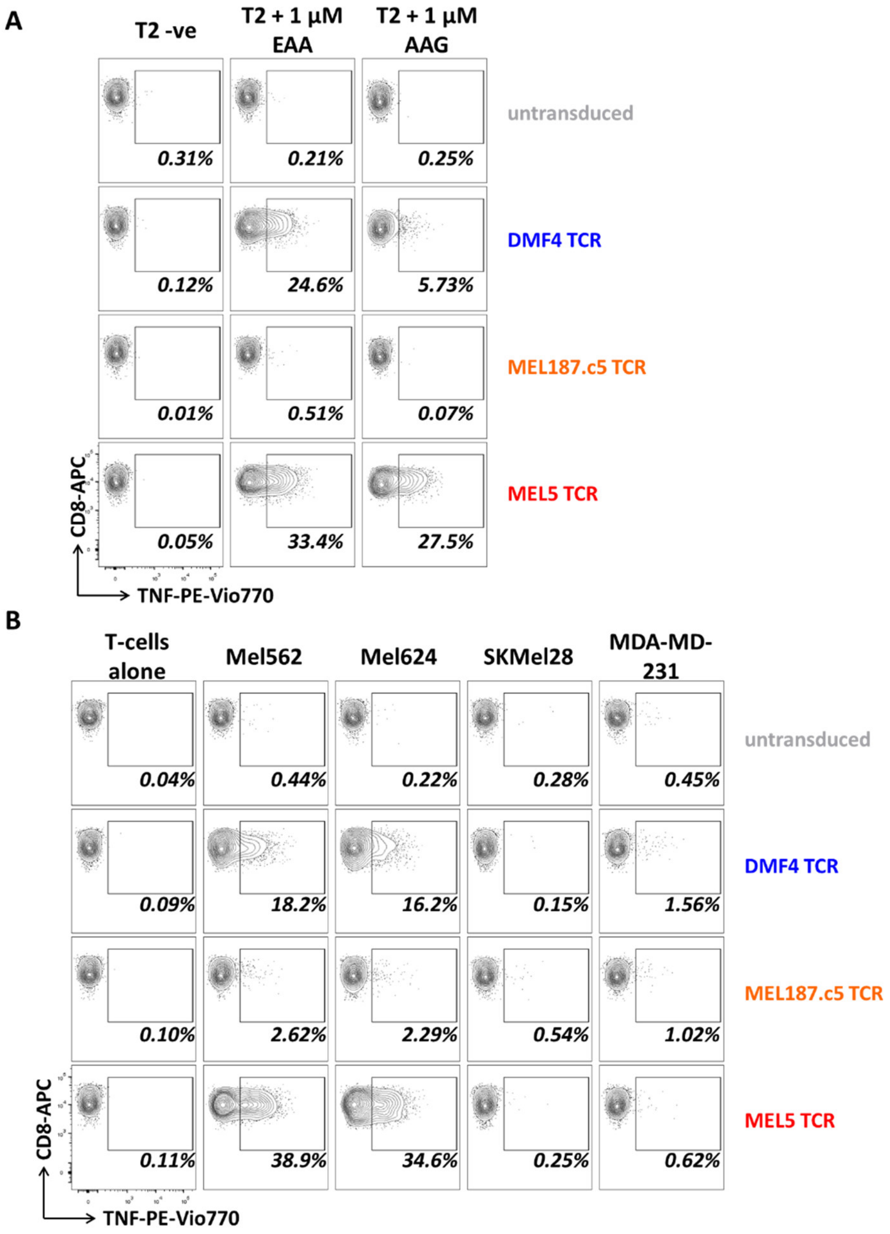

Supplementary Figure S1. TNF production in response to MART-1 decamer and nonamer peptides presented by T2 cell line (A) or endogenously processed and presented epitopes by cancer cell lines (B). Numbers on contour plots indicate the percentage of cells positive for TNF within viable CD3+CD8+ (and rat CD2+ where applicable) cells. Representative data, from experiments performed in duplicate, using PBMCs from three different donors, transduced with MEL5, MEL187.c5 or DMF4 TCRs, are shown.

2.2 Supplementary Figure S2. Functional assessment of the response towards HLA-A*02:01/MART-1 ELAGIGILTV mediated by DMF4, MEL187.c5 and MEL5.

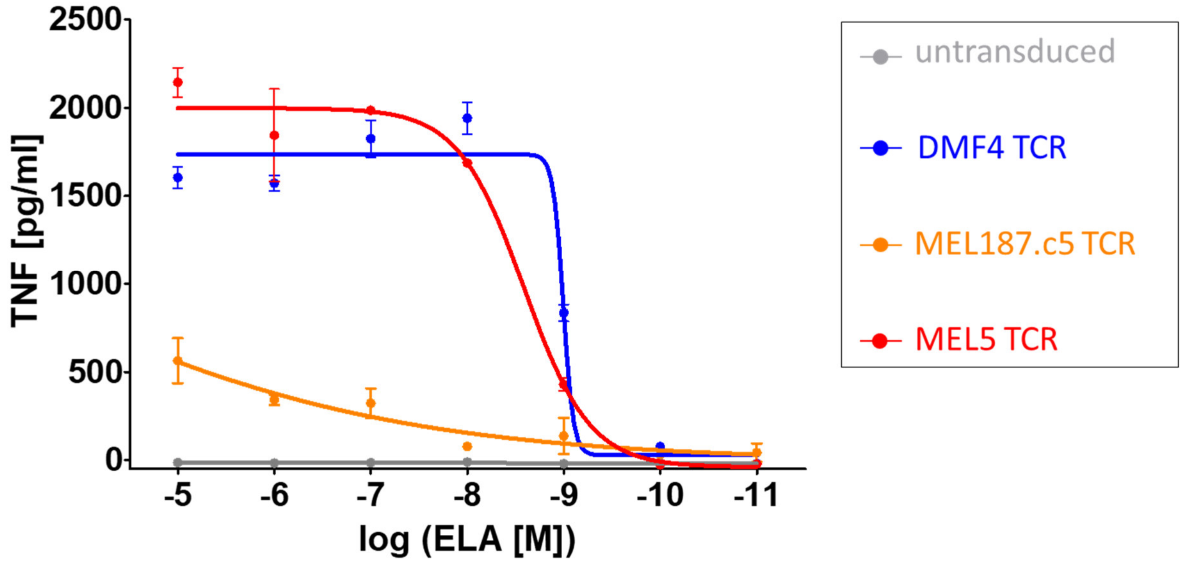

Supplementary Figure S2. TNF release in response to titrated concentrations of MART-1 heteroclytic peptide ELAGIGILTV. Quantification of secreted TNF was performed by ELISA. Experiments were performed in duplicate, using PBMCs from three different donors, transduced with MEL5, MEL187.c5 or DMF4 TCRs. Error bars denote standard error of the mean from two biological replicates (two independent experiments) using the TCR-transduced T-cells from one representative donor.

2.3 Supplementary Figure S3. Rigidity features of the AAG peptide in the MEL5-A2-AAG and DMF4-A2-AAG complexes presented using 'stripy plots' from FIRST

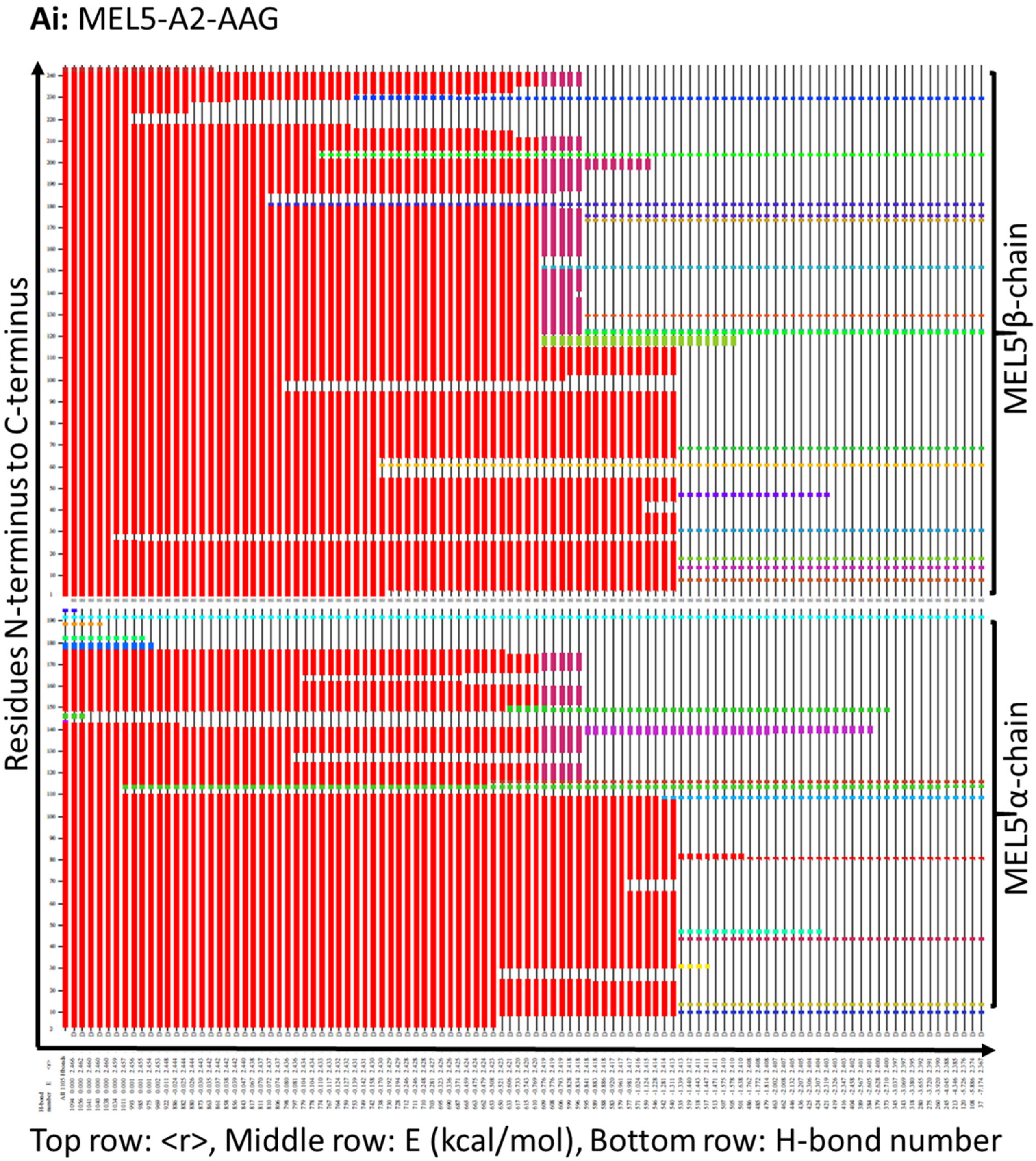

Aii: MEL5-A2-AAG

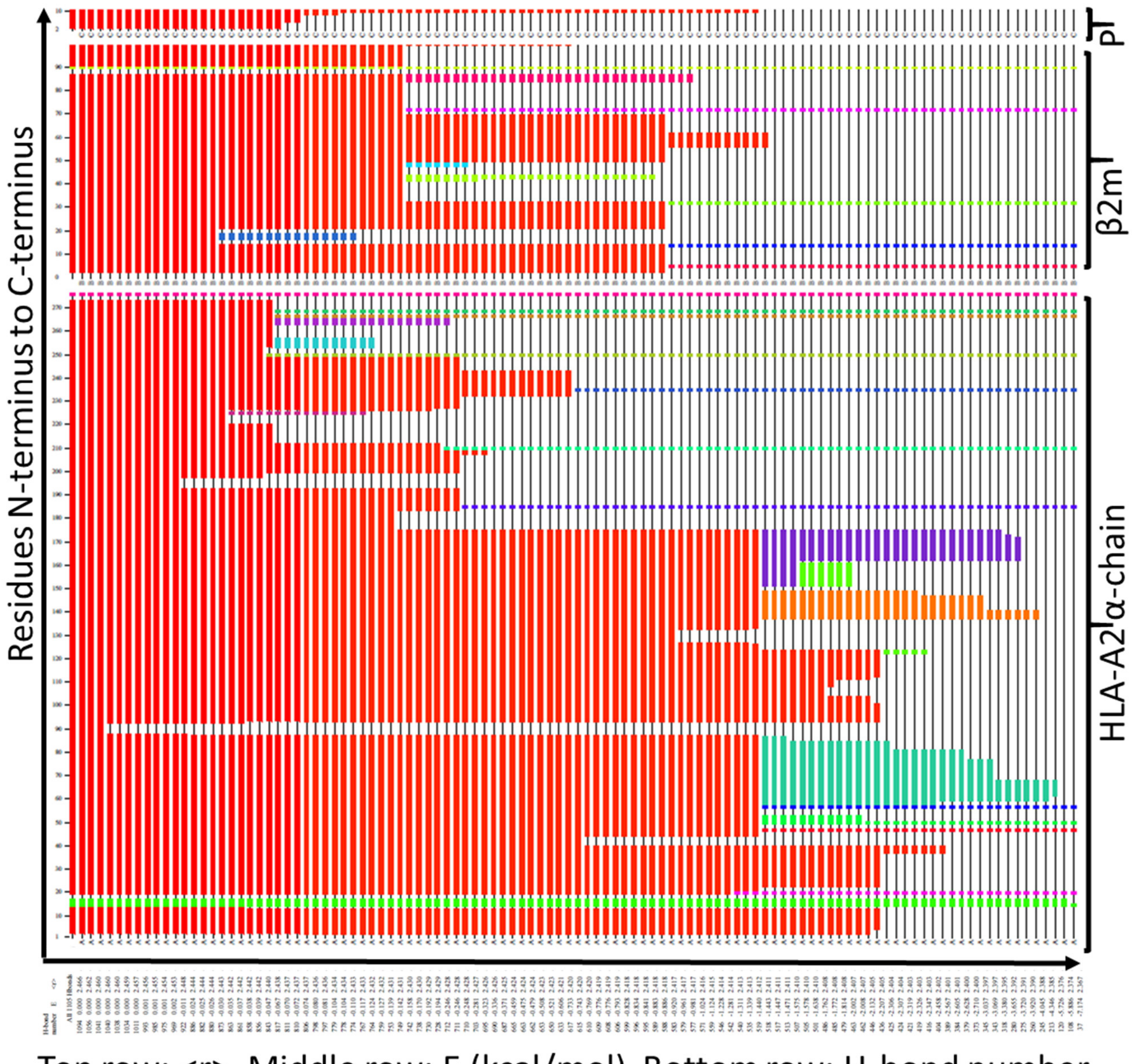

Top row: <r>, Middle row: E (kcal/mol), Bottom row: H-bond number

Bi: DMF4-A2-AAG

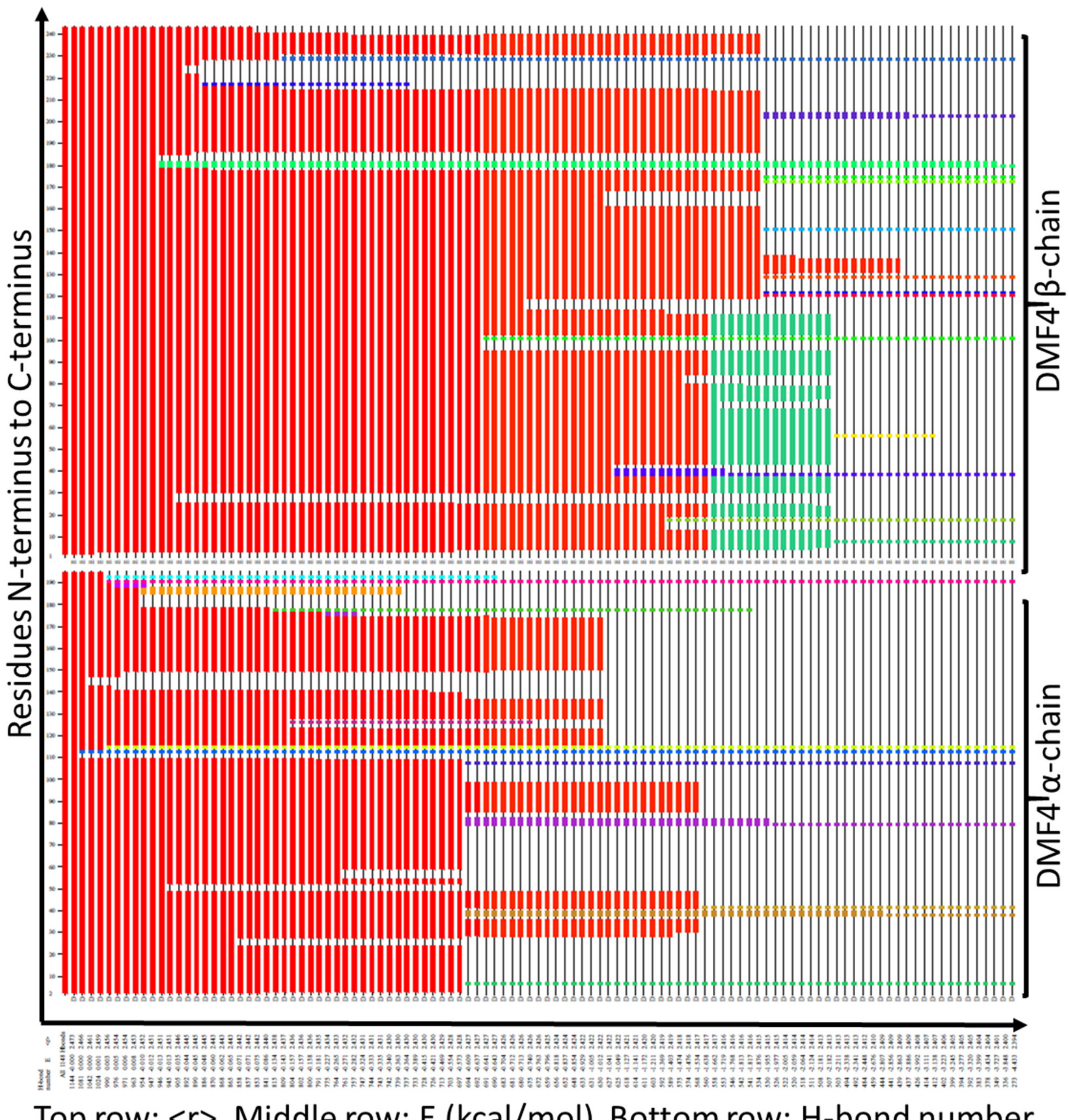

Top row: <r>, Middle row: E (kcal/mol), Bottom row: H-bond number

Bii: DMF4-A2-AAG

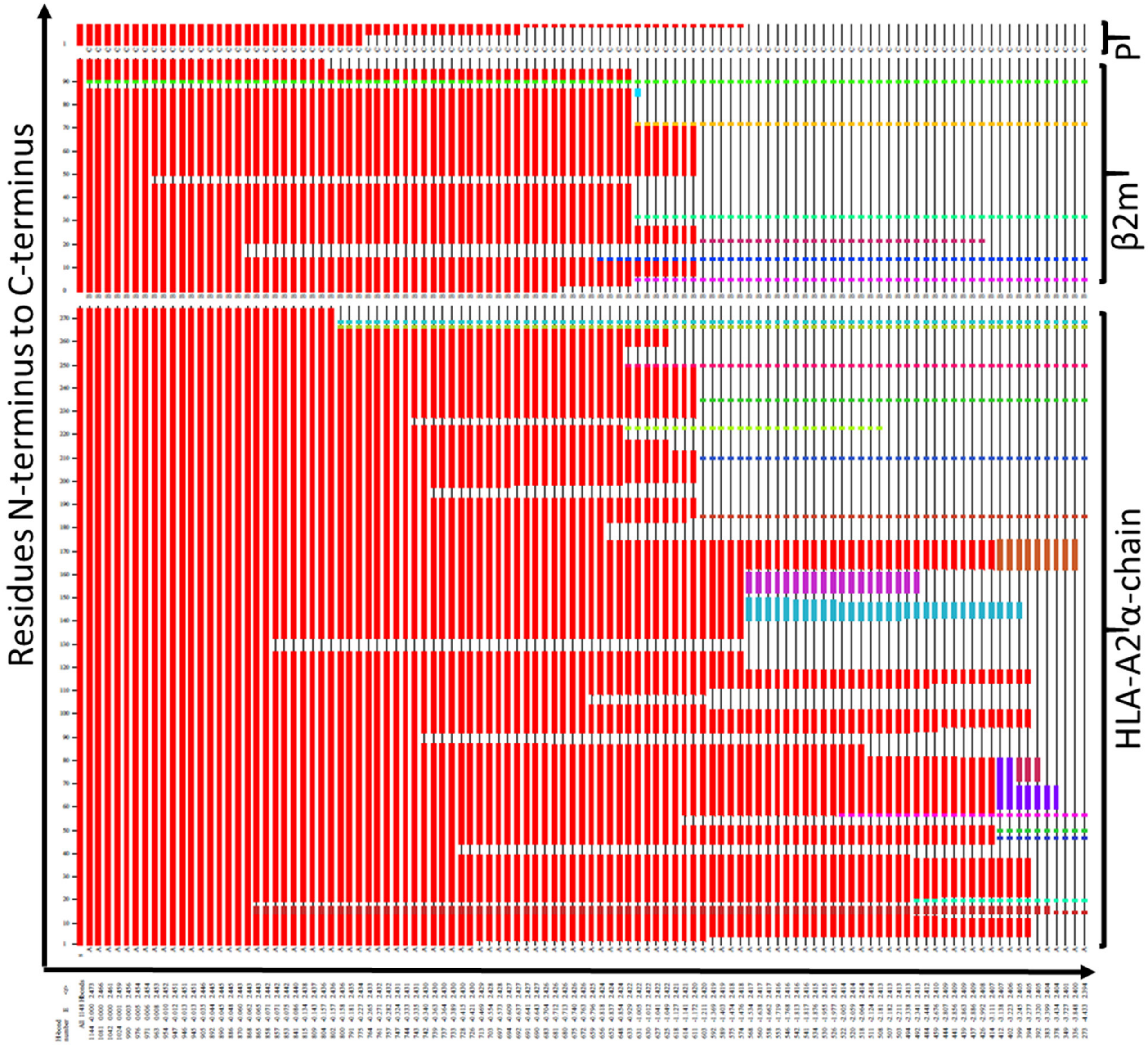

Top row: <r>, Middle row: E (kcal/mol), Bottom row: H-bond number

Supplementary Figure S3. FIRST dilution analysis of the mutual rigidity of the residues in the AAG peptide based on the distribution of constraints. The assessment was performed repeatedly while successively excluding hydrogen bonds, from weakest to strongest, by lowering an energy cutoff parameter. The “stripy plots” report the distribution of rigid clusters in the structure. A residue is described as “flexible” if it is not mutually rigid with any other residue. (**A**) Stripy plot analysis of the MEL5-A2-AAG structure. Peptide residue 2 was never mutually rigid within a rigid cluster. The remainder of the peptide (3-10) was detected as rigid with RC1, but only until the cutoff reached -0.070 kcal/mol, which is negligible. Residues 5-10 were rigid with RC1 only until a cutoff of -0.074 kcal/mol, likewise negligible, at which point residues 5-7 became flexible. Residues 8-10 were rigid with RC1 until a cutoff of -0.104 kcal/mol (barely significant) at which point residue 8 became flexible. Residues 9-10 were rigid with RC1 until a cutoff of -1.443 kcal/mol, and then became flexible. **(B)** Stripy plot analysis of the DMF4-A2-AAG structure. At the start of the rigidity dilution, the peptide was fully rigid (part of the largest rigid cluster, RC1, which initially encompasses almost the entire structure) until the cutoff dropped to -0.265 kcal/mol. The N terminal residues 1-4 then became flexible. Peptide residues 5-9 remained part of RC1 until a cutoff of -0.641 kcal/mol. Residues 5-7 then became flexible. The C terminal residues 8-9 remained part of RC1 until a cutoff of -1.534 kcal/mol. This reflects the strong hydrophobic interactions of this terminal with the HLA. At lower cutoffs the entire peptide was flexible. These data were used to generate **Figure 6C**. These data were generated from a single data set derived from X-ray crystallographic analysis.
